# Alcohol consumption, life history and extinction risk among Raute hunter–gatherers from Nepal

**DOI:** 10.1017/ehs.2024.42

**Published:** 2024-11-11

**Authors:** Inez Derkx, Gina Menn, Sudarshan Subedi, Nagendra Upadhyaya, Prajwal Rajbhandari, Anita Gyawali, Ruth Mace, Jaume Bertranpetit, Lucio Vinicius, Andrea Bamberg Migliano

**Affiliations:** 1 Department of Evolutionary Anthropology, University of Zurich, Zurich, Switzerland; 2 Tribhuvan University, Kathmandu, Nepal; 3 Committee to Study the Social, Cultural, Economic and Geographical Habitat of Raute Community, Nepal; 4 Research Institute for Bioscience and Biotechnology, Lalitpur, Nepal; 5 Department of Anthropology, University College London, London, UK; 6 Institut de Biologia Evolutiva (CSIC-Universitat Pompeu Fabra), Barcelona, Spain

**Keywords:** Hunter–gatherers, sedentarisation, Raute, Nepal, alcohol

## Abstract

Hunter–gatherer populations underwent a mass extinction in the Neolithic, and in present times face challenges such as explicit sedentarisation policies. An exception is in Nepal, where the nomadic Raute people receive monthly governmental individual payments. One consequence of the money transfers has been a significant increase in alcohol consumption, with nearly all individuals drinking industrially produced alcohol. Here we investigate the Raute demography based on a full census of 144 individuals. We show that the Raute exhibit the short life expectancies typical of other hunter–gatherer populations from Africa, Asia and America. Bayesian survival trajectory analysis demonstrated that heavy drinking by either parent substantially reduces offspring survival to age 15. Bayesian regressions revealed a significant effect of heavy drinking on maternal fertility by decreasing the number of living children and reducing the proportion of live children at the end of maternal reproductive life. Although the absence of data prior to monetary support precludes a direct assessment of long-term demographic trends, relatively stable population sizes over the last decades and a fertility rate close to the replacement rate rule out an imminent population crash. Further studies are required to elucidate the Rautes’ origins and relationship with other nomadic people in the region.

## Graphical abstract

**Figure:**
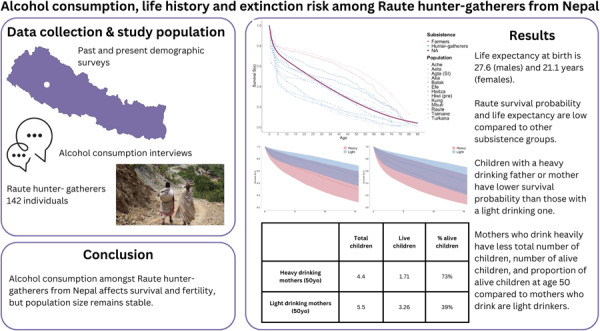

**Social media summary:** Alcohol consumption amongst Raute hunter–gatherers from Nepal affects survival and fertility, but the population size remains stable.

## Introduction

Hunter–gatherer populations have undergone continuous extinction since the beginnings of the Neolithic period (Zhang & Mace, 2021). Over recent decades, the few extant hunter–gatherer groups have faced further challenges to their social and ecological environments as a result of further expansion of farming and cities, market integration, climate change and other factors (Froment, 2001; Hitchcock et al., 2020). Lifestyle transition has been shown to affect hunter–gatherer demography and life history strategies in different ways. Pressure from neighbouring farming or urban groups has often led to outright population decrease in both the distant and the recent past, as exemplified by the extinction of the vast majority of hunter–gatherer groups previously occupying areas from large continental areas territories as Australia (Bianco & Rhydwen, 2001; Pearson, 2001) to smaller territories such as the Andaman Islands (Thangaraj et al., 2003; Venkateswar, 1999). Social deterioration has also been widely documented, as in the case of some Evenki hunter–gatherer groups in China where around 40% of deaths recorded in the 1960s were caused by accidents and suicide (Dong, 2015). Alternatively, groups may undergo sedentarisation and loss of the hunter–gatherer lifestyles but survive as ethnic or cultural groups (Fa et al., 2021; Hill & Hurtado, 2017; Headland et al., 2011; Lee, 1972; Loung, 1959; Paulin, 2007). In such cases, formerly hunting and gathering groups often exhibit changes in life history and reproductive strategies. For example, among the Agta people from the Philippines, dietary shifts and sedentarisation of some households and camps were associated with a drastic increase in infant mortality compared with more nomadic Agta mothers, owing to increased levels of communicable diseases and population density (Page et al., 2016). However, sedentarised Agta mothers also exhibited a faster life history strategy characterised by increased fertility and reduced mean parental investment per child that compensated for increased mortality rates, leading to an overall increase in reproductive success. A fast life history strategy seems to be a plastic and behavioural adaptation to hunter–gatherers exposed both to new carbohydrate-rich food items (rice from neighbouring farmers) and increases in (especially infant) mortality (Page et al., 2016, 2024).

The above suggests that sedentarisation and market integration may have both negative and positive demographic effects on hunter–gatherer populations. While money and trade may, for example, reduce food uncertainty through the purchase of carbohydrate-rich foods and provide access to medicines, they may also have negative effects on health and fertility (Donders & Barriocanal, 2020; Dounias & Froment, 2006, 2011; Froment, 2001; Leepile et al., 2021). In particular, the introduction of industrially produced alcohol has had a deleterious effect on many hunter–gatherer societies (Knight et al., 2021; Oishi & Hayashi, 2014; Ramirez Rozzi, 2018; Koirala et al., 2023). Immediate and long-term consequences of increased alcohol consumption such as accidental deaths, sexual abuse, suicide and homicide have been widely reported (Calabria et al., 2010; Coetzee et al., 1994; Gallois et al., 2021; Lehti et al., 2009; Long et al., 2013; Seale et al., 2002; Townsend, 2016; van der Westhuyzen et al., 1987). For example, 23% of recorded deaths among the Reindeer-herding Sami in the late 1990s were due to suicide, and 27% were due to road or snowmobile accidents, with alcohol abuse found in 15% of cases (Ahlm et al., 2010). Alcohol was associated with an increase in violence and accidental deaths among the Hadza (Marlowe, 2010) and the Canadian Inuit (Seale et al., 2006).

The short- and long-term effects of alcohol consumption on human health, fertility and overall survival have been extensively studied (Room et al., 2005). Alcohol dependence has been linked to delayed reproductive onset and increased risk of infertility, and even moderate drinking behaviour can have a negative impact on fecundity (Eggert et al., 2004; Jensen et al., 1998; Waldron et al., 2008). In Western populations, prenatal exposure to alcohol may lead to lower sperm concentration, while chronic alcoholism has been linked to decreased testosterone, semen volume, sperm count, motility and number of morphologically normal sperm cells (de Angelis et al., 2020; Muthusami & Chinnaswamy, 2005; Ramlau-Hansen et al., 2010). Child behavioural impairments such as sleep disorders and irritability resulting from prenatal alcohol exposure can also increase child mortality, while gestational alcohol consumption may lead to an increased risk of postnatal consumption, potentially leading to a higher risk of neglect, abuse or insufficient responses when infants are ill (Burd & Wilson, 2004). Indigenous communities might be even more prone to suffering from the effects of alcohol consumption; for instance, the death records of American Indians and Alaska Natives between 1999 and 2009 showed higher rates of alcohol-attributable death compared with non-hispanic Whites, while reduced fertility owing to alcohol consumption was identified in a Baka population in Le Bosquet, Cameroon (Landen et al., 2014; Ramirez Rozzi, 2018).

Explicit sedentarisation policies have been actively promoted in the Philippines, Namibia and India, amongst other countries, potentially inducing the same combination of positive and negative effects on the remaining nomadic populations. An exception to compulsory sedentarisation policies is in Nepal, where governments have attempted over the last few decades to support a handful of ethnic minority populations identified as being under risk of extinction (Asian Development Bank, 2011) by providing a monthly per capita money allowance. The least known of such groups is possibly the Raute, a small population from Western Nepal and regarded as one of the last nomads in the Himalayas (Fortier, 2009; Singh, 1997). Very little is known about the origins of the Raute and their relationships with other nomadic people from India and Nepal, as no genetic study of the group exists, no written or oral history exists, and they claim not to know their ancestry beyond four generations. The only comprehensive ethnography of the Raute is the monograph by Fortier (2009), providing most of the information we mention in our article. Although they are aware of other, settled, people in western Nepal also carrying the name Raute, the Raute do not see them as a related group but instead as villagers and landowners (Fortier, 2009). Historically, they have made a living hunting monkeys and gathering wild plants and fruits from the surrounding forests, whilst trading self-produced woodware for dry staples such as rice. This has traditionally distinguished them from other locals in the region, who mostly engage in wage labour and small-scale agriculture and whose children attend schools. Some limited conflict has been recorded, for example owing to the Raute sometimes cutting trees planted by surrounding communities as part of a governmental reforestation effort started in the 1990s (Adhikari et al., 2007). Although the Raute have contact with villagers mostly for trade, they practise strict endogamy and do not intermarry outside of their nomadic group.

**Figure 1.**
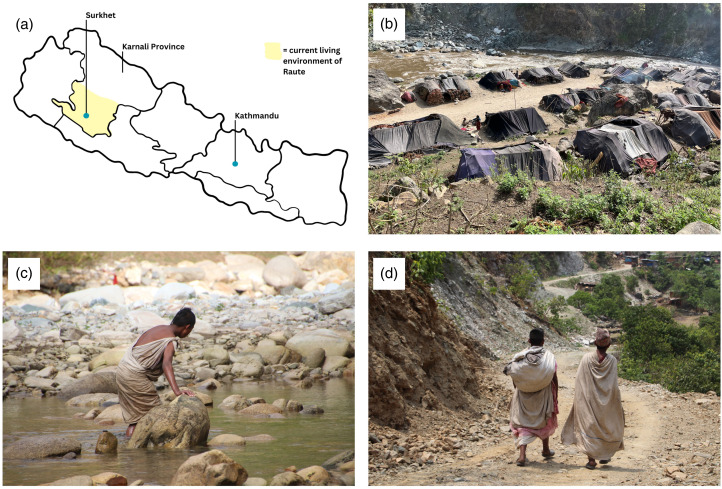
Range, distribution, and residential camps of the Raute from Nepal. (a) Map of Nepal and current range of Raute in Western Nepal. (b) Raute residential camp in Jajarkot, Karnali province, Nepal. (c) Fishing using nets. (d) Two Raute carrying fishing nets. Photos taken in May 2022 by Gina Menn.

Crucially, 2009 was also the year when the Raute started to receive a monetary allowance (500 rupees) from the Nepali government under its social security programme for endangered ethnic groups, increased in 2016/2017 to 2000 rupees and currently 4000 per month per head. As discussed above, monetisation and market integration often have significant effects on the demography and health of hunter–gatherer populations, which justifies the need for an update on the current status of the Raute. In 2022, we visited the Raute who at that point consisted of 144 individuals residing in the Jajarkot district, Karnali province, Nepal (Fig. 1). In the past they were described as occupying areas of high altitude in the Himalayas of up to 1500 m in the summer, and never below 600 m in winter (Bista, 1976), although during our field trip we observed that now they may be found at higher elevations. The Raute remain highly mobile, but while an earlier ethnography reported that they would never stay for longer than three weeks in the same location (Reinhard, 1974), our estimate is now closer to three months. We found them divided into approximately 40 households and residing in tents held together with wood and cloth and rebuilt after every move. Currently the Raute receive material aid from various organisations in the form of clothing, blankets and cloth for the tents. Nonetheless, the Raute remain culturally and demographically isolated from neighbouring populations, with not a single instance of immigration, emigration or inter-marriage. Furthermore, they do not consider themselves to be part of the same groups also calling themselves ‘Raute’ and living in the Dadeldhura and Darchula districts (Fortier, 2009).

While individuals still explore forest resources, the money handouts allow the Raute to purchase agricultural products, medicines and other goods. However, in addition to food security and other benefits, money transfers seem to have enabled the consumption of low-quality commercial alcohol. A recent study by Koirala et al. (2023) showed that hypertension was more prevalent amongst Raute self-reported drinkers compared with non-drinkers, and that both the Raute and neighbouring villages were aware of alcohol-related changes in their behaviour and health. It is not clear whether money transfers have produced a net benefit or cost to the Raute's demography, both because there are no reliable data on survival and fertility in the population before or after the start of the money transfers, and because there is no study of the effects of alcohol consumption on those parameters. What can be said on the basis of limited previous reports is that the Raute population seems to have remained relatively stable over the last 50 years, ruling out either a current population boom or imminent extinction (Supplementary Table S1). Reinhard (1974) counted 35–36 huts and hence estimated a population size of between 105 and 140 individuals. Singh (1997) counted 130 individuals (67 females) and Fortier (2000, 2009) reported about 150 individuals and between 39 and 43 tents. A government census in 2014 and continuous documentation since 2018 have recorded population size more reliably, reporting 144 individuals in 2022 when our data were collected. In the following, we present the first estimate of life expectancy in the Raute and comparisons with survival curves in other hunter–gatherer and small-scale populations, a report on levels of alcohol consumption; an assessment of the effects of alcohol use by parents on offspring survival and the relationship between alcohol consumption and female-specific fertility in Raute women.

## Methods

### Ethics statement

Our research was approved by the ethics commission of the University of Zurich (ethics code no. 20.2.8) and performed in agreement with the government of the Karnali province in Nepal and the three Raute leaders. All participants provided informed consent after an initial group demonstration, explanation of research procedures and further individual consultation in Nepali. Participants and households were individually given a small compensation (mostly in the form of toiletries and cooking items and as a token of gratitude for their collaboration) and were told that they could withdraw their consent at any point. All data were anonymised.

### Ethnographic data collection

Ethnographic data collection took place during two field trips between May and June 2022 and in December 2022, during which we censused the entire population of 144 individuals (70 males and 74 females). All interviews and procedures were performed in Nepali with the help of local field assistants We collected information on sex, age, group composition, household composition,and kinship, and performed in-depth interviews with 13 Raute elders about their lifestyle and lifestyle changes in the last few decades. Later, we performed short interviews with 115 individuals (52 females, 63 males; 66 adults and 49 children under 18) aged 4 and above, during which they were asked about their alcohol consumption, frequency and rationales for drinking. Later, 60 individuals were assessed in more detail regarding their alcohol consumption patterns, including six individuals not included in the short interviews. Lastly, 56 women aged 8–82 were interviewed about their reproductive history in a private setting by a female interviewer, 42 of whom also participated in alcohol consumption interviews.

### Population censuses

Population demographics have been continuously recorded since 2018 by local field workers, who document all births and deaths alongside information such as the relations of the individual, date of birth, date of death and cause of death. In addition, the Karnali province performed a population census in 2014, in which 141 individuals (66 females, 75 males) were present. From those, at least 22 individuals had died, 114 individuals were still alive, and at least 30 individuals had been born, resulting in a population of 144 individuals in 2022. Ages reported in 2014 and in the annual censuses from 2018 were compared to eliminate discrepancies and check for age heaping and other artefacts. Age estimates between 2014 and 2022 showed an average discrepancy of only 0.9 years, which is partially expected since age is reported in full years, and the two censuses were collected in different months.

### Age-specific survival and life expectancy estimation

Owing to the small population size and a high number of right-censored cases (live individuals), we computed age-specific survival with the *R* package *BaSTA* for Bayesian survival trajectory analysis (v1.9.5; Colchero et al., 2012; see Vinicius et al. 2014 for technical details) assuming a Siler (U-shaped) mortality pattern typical of hunter–gatherer and other human populations with high infant mortality (Finch, 1990). BaSTA implements a Bayesian framework to estimate age-specific survival curves using capture–recapture or recovery data (Colchero et al., 2012). A sample of 187 individuals born between 1931 and 2021 was selected based on the availability of recorded birth and death dates. For each of the 15 people alive in 2014 and dead when the censuses were resumed in 2018, but with unknown death dates, we randomly selected a value between 2014 and 2018. The resulting survival curve *S*(*x*) represents the age-specific decrease in the probability of survival and is equivalent to the *l*(*x*) variable commonly used in life tables presenting mortality and survival probabilities as a function of age. We calculated e(*x*), or life expectancy at age *x* for males (*n* = 97) and females (*n* = 90) with the unpublished package *paramDemo* (v1.0.0; Colchero, 2023) and *BaSTA* with sex or drinking levels as predictors.

We compared the age-specific survival probabilities and life expectancy in the Raute with a set of 13 hunter–gatherer and small-scale societies. Published life tables were available for the !Kung (Howell, 1979), Ache (Hill & Hurtado, 2017), Hadza (Blurton-Jones, 2016), Hiwi (Hill et al., 2007; pre- and post-contact), Tsimane (Gurven & Kaplan, 2007) and Yanomamo (Neel & Weiss, 1975). For the Aeta, Aka, Batak, Efe, Mbuti and Turkana we used Weiss model life tables (Migliano, 2005; Migliano et al., 2007). Whenever available, we used sex-specific data. For life expectancy estimation, we compared life expectancy at ages 0, 5, 10, 15 and 50 years for males and females separately whenever sex-specific information was available.

### Child survival and parental alcohol drinking

We used three *BaSTA* models for a subsample including only individuals aged 15 years and younger (*n* = 76, 58 alive and 18 dead) to estimate age-specific survival for children. The models estimated how maternal and paternal frequency of drinking influenced the survival trajectory of their children. The subset includes only children with information available on the drinking habits of their mothers (*n* = 32, 24 alive and eight dead) and fathers (*n* = 56, 46 alive and 10 dead). Information about drinking frequency (in units of drinks per day) was collected during the interviews. One drink was defined as one bottle or large cup of a local commercial rice wine (300 ml, and with 17.5% alcohol content), corresponding to approximately four standard units of alcohol. There was no stigma around alcohol use and participants were interviewed privately to increase the reliability of self-report. Participants were then divided into non-drinkers (no drinks per day), light drinkers (one to two drinks per day for females, one to three drinks per day for males) and heavy drinkers (three or more drinks per day for females, four or more drinks per day for males).

### Female fertility and frequency of drinking alcohol

We investigated how frequency of drinking alcohol impacted the total number of children, number of living children and proportion of children alive for 42 women with known reproductive history. We applied Bayesian Poisson regressions to predict total number of children and number of living children, and binomial regressions to predict the proportion of children alive, both implemented in the *brms* package (v2.19.0; Bürkner, 2017) in R. All regressions used default priors and included an interaction factor of age with frequency of drinking.

## Results

### Survival and life expectancy in the Raute are typical of other hunter–gatherer populations

**Figure 2.**
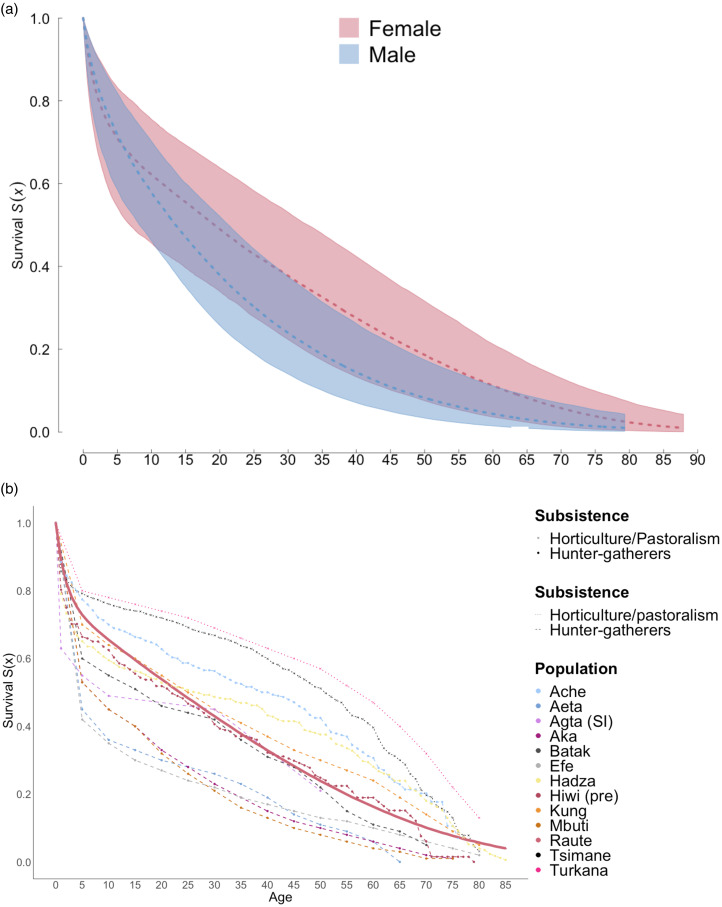
Survival of Raute hunter–gatherers. (a) Age-specific survival curve *S*(*x*) for males and females. (b) Overall comparison to other populations. Data for other populations are from the sources cited in Table 1.

Our survival models produced estimates of life expectancy at birth of 27.6 years for females and 21.1 years for males (Fig. 2a), which is higher than that in Central African and Southeast Asia hunter–gatherers, but much shorter compared with other hunter–gatherers such as the Hadza, San (!Kung) and Ache (Fig. 2b). Life expectancy was higher by age 5 but then dropped more sharply compared with the other groups (Table 1). Although all populations experience a drop in age-specific survival, the Raute show a starker decrease up to the age of around 45 compared with the other hunter–gatherer populations. As with other nomadic populations, the Raute also show shorter lifespans compared with horticulturalist and pastoralist groups. It should also be mentioned that the broad 95% credible intervals were caused by the small sample size.

**Table 1.** Life expectancy e(*x*) at various ages in the Raute and other societies: !Kung (Howell, 1979), Ache (Hill & Hurtado, 2017), Hadza (Blurton-Jones, 2016), Hiwi (Hill et al., 2007; pre- and post-contact), Tsimane (Gurven & Kaplan, 2007), Yanomamo (Neel & Weiss, 1975), Aeta, Aka, Batak, Efe, Mbuti and Turkana (Migliano, 2005; Migliano et al., 2007)

Subsistence	Population	e(0)		e(5)		e(10)		e(15)		e(50)	
		Female	Male	Female	Male	Female	Male	Female	Male	Female	Male
Hunting and gathering	Mbuti	15.6		23.9		22.3		20		12.1	
	Efe	16		31.7		32.5		32.5		18.1	
	Aeta	16.5		28.5		30.6		27.3		9.4	
	Aka	16.6		25.8		24.5		22.5		13.3	
	Batak	24.2		34.5		32.5		29.5		12.1	
	**Raute**	**27**.**6**	**21**.**1**	**31**.**8**	**22**.**5**	**30**.**2**	**21**.**7**	**28**.**2**	**20**.**9**	**15**.**8**	**15**.**2**
	Hiwi (pre)	27.6	27.9	37.4	34.8	33.5	32.6	33.1	31.1	17.7	12.8
	Hiwi (post)	27.1	31.3	49.2	45.5	45.1	41.6	42.6	39.1	20.7	15.4
	!Kung	31.3		39		37.7		35		19.1	
	Hadza	35.6	30.8	48.1	42.1	47.0	41.2	44.8	38.2	20.5	15.7
	Ache	37.1	37.8	44.9	41.5	45.8	39.8	43.3	36.8	19.2	14.8
Farmers and pastoralists	Tsimane	42.8	44.2	49.8	49.4	46.7	46.2	42.6	42.7	18.4	16.7
	Turkana	47.5		54.4		50.7		46.6		21.1	

At age 15, Raute males and females have lower remaining life expectancy than all groups apart from some of the Central African hunter–gatherers, with a value of 28.2 years for females and 20.8 years for males, but by the age of 50 the curves resume an intermediate position compared with other hunter–gatherers (remaining life expectancy of 15.8 years in women, and 15.2 years in men). Overall, data from the Raute point to survival curves compatible with other hunter–gatherer populations, with relatively short lifespans at birth, at age 15 near sexual maturity and at age 50 when compared to horticultural and pastoralist populations.

### The Raute report various reasons for alcohol consumption

**Figure 3.**
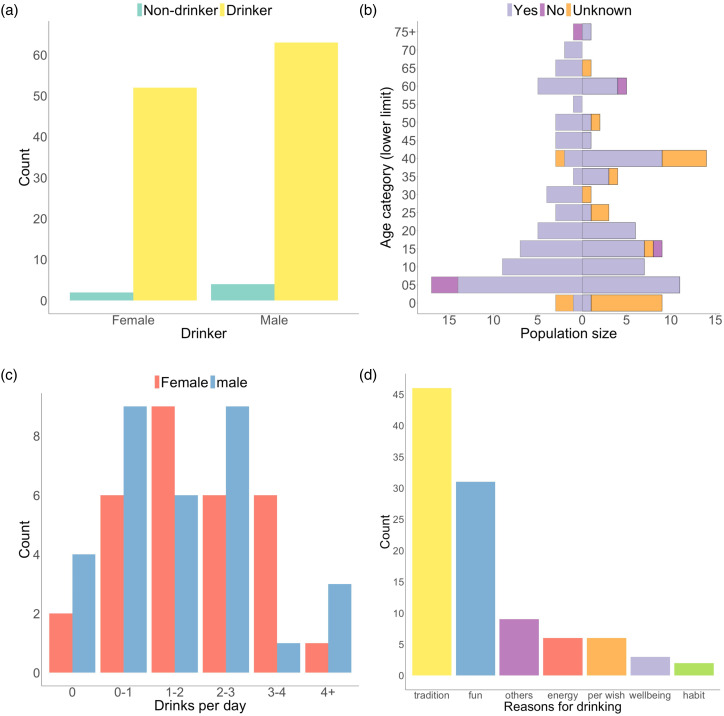
Patterns of alcohol consumption among the Raute. (a) Drinking by sex. (b) Population pyramid (males on the left, females on the right), with added information on drinking status (yes, no, or unknown) by age and sex. (c) Drinking frequency by sex. (d) Reported reasons for alcohol consumption.

Next, we examined patterns of alcohol consumption in the Raute. Out of 121 participants or 85% of the population, only six (5%) reported not drinking alcohol (Fig. 3a). There was no statistically significant relationship between being a drinker and either sex (Fisher test: *p* = 0.691) or age (Wilcoxon test: *W* = 290.5, *p* = 0.519). The youngest drinker was only 4 years old, while the youngest non-drinker was 5 years old (Fig. 3b), demonstrating a very early onset of alcohol consumption. Drinking frequency varies by sex (Fisher test: *p* = 0.047), with most individuals consuming between one and three drinks per day (Fig. 3c).

The Raute mainly mentioned tradition and fun as reasons for drinking (Fig. 3d). With regard to the cultural role of alcohol, older Raute people indicated that only the consumption of homemade, traditional rice wine stems from their culture and religion. They also indicated that when they were young the only alcoholic drink was traditional rice wine, which was not consumed by all individuals. Others stated that alcohol plays no role in their culture and is now consumed out of individual habit. This suggests that the current widespread consumption of industrially produced alcohol has no deep historical roots, while the more traditional consumption of rice wine was never adopted by all members of the population.

### Alcohol consumption negatively impacts offspring survival

**Figure 4.**
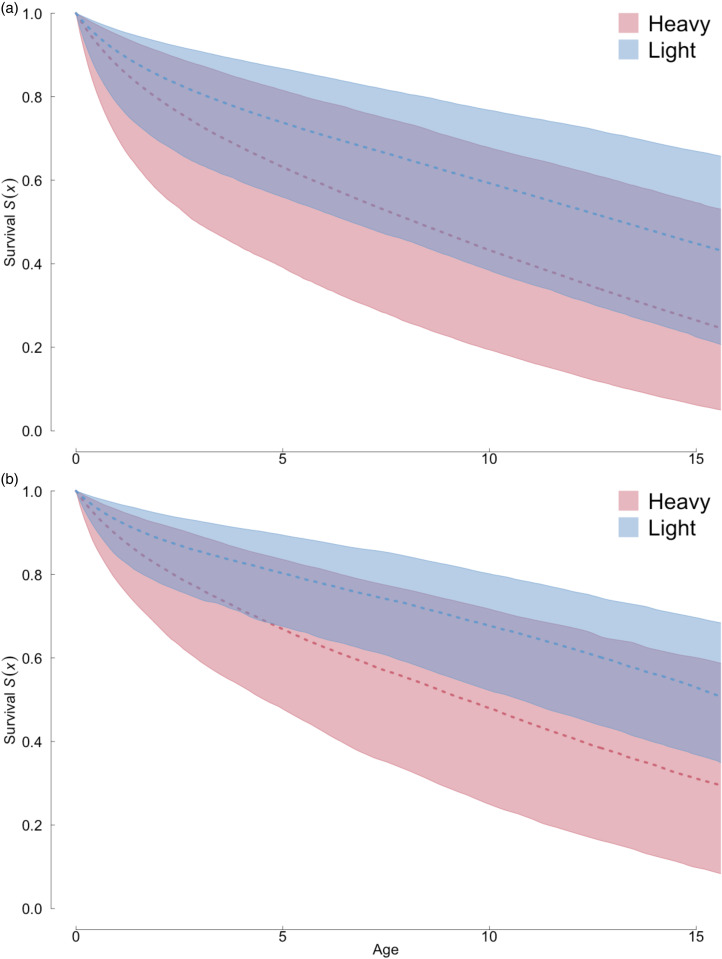
Effects of levels of alcohol consumption on child survival in the Raute. (a) Maternal effects. (b) Paternal effects. Survival curves show survival probability *S*(*x*) of offspring to age 15.

The nearly universal and frequent consumption of purchased alcohol suggested a possible effect of drinking on survival and mortality. A direct comparison between the survival of drinkers and non-drinkers proved elusive, since virtually all individuals were classified as alcohol drinkers (out of six non-drinkers, only two were adults). We therefore examined whether survival of children to age 15 was influenced by the level of alcohol consumption (light vs. heavy) by their mother (20 light drinkers, 12 heavy drinkers), father (41 light drinkers, 15 heavy drinkers) or any parent (13 couples with one heavy drinker, 10 couples with no heavy drinker). Figure 4 shows that having either a paternal or maternal heavy drinker significantly reduces the survival of children to age 15, with respectively *S*(15) = 0.55 and *S*(15) = 0.30 for light- and heavy-drinking mothers (Fig. 4a), and *S*(15) = 0.59 and *S*(15) = 0.40 for light- and heavy-drinking fathers (Fig. 4b). Overall, the results show that higher drinking levels by either parent reduce offspring survival to adulthood.

### Alcohol consumption reduces overall fertility of Raute women

Next, we examined the effects of drinking levels on Raute fertility. First, we applied a Bayesian Poisson regression to predict the number of maternal live children from age and drinking level. The regression revealed the expected increase in the number of living children per mother with age, and a significant and negative interaction between age and heavy drinking, predicting a lower number of live children in heavy drinkers from the age of around 36.5 years. At the age of 50 around the end of maternal reproductive lives, while heavy drinkers are predicted to have 1.65 live children, the value for light drinkers is almost double at 3.2.

A regression of the number of total children (dead and alive) on maternal age and drinking level has shown a similar pattern, and predicted values of 5.51 total children for light drinkers and 4.4 for heavy drinkers at age 50. In contrast, in a regression on the number of dead children, while age remains a meaningful predictor, drinking levels have no effect (mean coefficient estimate of 0.27; 95% credible interval [−0.95, 1.58], with 33.7 and 66.3% of the posterior distribution of coefficients below and above zero respectively). Finally, a binomial regression predicting the proportion of live children showed increasing survival for light drinkers but the opposite for heavy drinkers, so that 78% of children are predicted to be alive when a light-drinking mother is 50 years old, and only 42% for heavy drinkers. Overall, the results indicate that female heavy drinkers produce fewer living descendants by the end of their reproductive lives. This occurs because of an overall reduction in fertility (total number of children) in heavy drinkers compared with light drinkers, rather than owing to an increase in the number of dead offspring.

## Discussion

Our survival and life expectancy comparisons across populations showed that the Raute exhibit the short life expectancies typical of other hunter–gatherer groups, and also shorter than in some horticultural or pastoralist groups. In addition, we also showed that heavy drinking correlates with a noticeable reduction in offspring survival probability to only 55% at age 15. This implies that at least heavy drinkers are already facing fitness costs owing to alcohol consumption. This may also be true for light drinkers (whose offspring have a predicted survival rate of 70% at age 15), although a comparison with non-drinkers was not possible. A sample of non-drinkers would have provided a measure of the overall effect of money transfers on Raute survival and fertility since 2008, without the negative consequences of alcohol.

Despite the fitness costs of alcohol that we identified, we cannot reliably predict demographic trends in the Raute based on available evidence. To properly estimate the overall effect of money transfers on population size, we would need to compare our data with survival and fertility data predating 2008, which are not available. We can, however, make two statements based on the relative stability of population size, which barely changed from the 150 people reported by Fortier (2009) to 144 in our census from 2022. First, short-term extinction in the next few years or decades does not seem to be imminent or inevitable. In fact, the total number of living offspring by the end of reproductive life across all Raute women (nearly two children, intermediate between the values from heavy and light drinkers) is not far from the replacement rate and may explain why the Raute have maintained very low population sizes over the past decades. Thus, extinction owing to a potential further increase in the levels and frequency of heavy drinking is only one of various possible scenarios that must be assessed in future studies but cannot be directly derived from our results. And second, money transfers are likely to also have positive effects seemingly offsetting the negative effects of alcohol on fitness, possibly in the form of more food security through the purchase of rice and other sources of carbohydrate or reduction in mortality through the purchase of medicines, among other factors that we cannot assess owing to a lack of comparative data prior to 2008. Our interviews and fieldwork observations are, however, compatible with a possible balance of effects. While we observed extensive drinking periods (sometimes over many days) after the dates of money distribution, several Raute elders also mentioned that they have less need to hunt now as most of their needs (food and money) are provided by the government, that they enjoy the extra free time and that life is easier than before.

Our findings have implications for policy and anthropology. A first conclusion is that despite the spread of alcoholism in the population, the money transfer may have unmeasured benefits. Given that there is no ongoing population crash in the Raute, and since local authorities now maintain continuous contact with the population, awareness campaigns and interventions from officials and charities may possibly help to address the problem of alcohol use in adults and children in the near future. From an anthropological perspective, our study represents a first assessment of survival and fertility in one of the few remaining hunter–gatherer groups in the Himalayan region. The Raute are a unique population whose relationship with other nomadic people in Nepal and India remains elusive. Future studies should further investigate the potential demographic consequences of alcohol consumption and other changes resulting from money transfers and incorporate the Raute into genetic and linguistic comparative analyses to possibly elucidate the origins and evolutionary relationships of this population.

## Supporting information

Derkx et al. supplementary material Derkx et al. supplementary material

## Supplementary material

The supplementary material for this article can be found at https://doi.org/10.1017/ehs.2024.42.

## Research transparency and reproducibility

Population-level demographic information and individual data about alcohol consumption are available on request from ABM. Individual data are not publicly available to protect the privacy of participants. Source code is available on https://github.com/inezd24/Raute_demography.
