# Supplementary material for: Alcohol consumption, life history and extinction risk among Raute hunter–gatherers from Nepal
Source: Evol Hum Sci. 2024 Nov 11;6:e45. doi: 10.1017/ehs.2024.42 (PMC11658931; doi:10.1017/ehs.2024.42)
Supplement: Derkx et al. supplementary material [file S2513843X24000422sup001.docx]

**Alcohol consumption, life history and extinction risk among Raute hunter-gatherers from Nepal**

Inez Derkx^1^, Gina Menn^1^ , Sudarshan Subedi^2^, Lal Bahadur Khatri^3^, Nagendra Upadhyaya, Prajwal Rajbhandari^4^, Anita Gyawali^5^, Ruth Mace^6^, Jaume Bertranpetit^7^, Lucio Vinicius^1*^  and Andrea Bamberg Migliano^1^

**Supplementary file**

Supplementary Figure S1

Supplementary Table S1

| **Source** | **Number of units** | **Size** | **Condition** |
| --- | --- | --- | --- |
| Reinhard (1974) | 35-36 huts | 105-140 | Nomadic |
| Nepal 'Yatri' (1983) | 4 groups | 352 | Nomadic |
| Ministry of Labour and Social welfare (1984) | 2 settled groups, 1 nomadic group, 4 houses at Dinsi | 400+125-135 | Settled & Nomadic |
| Bist (1985) |  | 200 | Nomadic |
| National Population Census, 1991 |  | 2878 | Raute |
| CEDA (1991), A report submitted to National Planning Commission |  | 375 | Raute |
| CERID (1991) Report based on National Population Census, 1991 |  | 2878 | Raute |
| An Analysis of the 1991 Population Census published by CBS (March, 1993) |  | 268 | Raute |
| Karki (Aug. 1993) | 60 families | 350 | Nomadic |
| Misra (Jan, 1993) | Many groups | 250 | Nomadic |
| Sanjyal (March, 1993) |  | 250 | Nomadic |
| Nepal (July, 1993) |  | 250 | Nomadic |
| Majhi (July, 1993) | one group | 300 | Nomadic |
| Nanda Bahadur Singh, 1997 | 37 families | 130 (63M, 67F) | Nomadic |
| Fortier (2000) | 39-43 tents | 117-172, +- 150 | Nomadic |
| Fortier (2009) |  | 150 more or less | Nomadic |
| Bista (1976) | 22 sheds |  | Nomadic |
| Statistical Pocket Book Nepal, 2008 |  | 658 (346M, 312F) | both? |
| Government/NGO census 2014* |  | 143 | Nomadic |
| Paudel (2016)* | 39 tents/sheds | 148 (83M, 65F) | Nomadic |
| Yasin (2017)* | 45 households | 162 (90M, 70F) | Nomadic |
| UZH, 2022 May* | 40 houses | 142 | Nomadic |
| UZH, 2022 December* |  | 142 | Nomadic |

**Table S1**. Overview of Raute population size estimates in the last 50 years. The table is adapted from Singh (1997) supplemented with new data retrieved in the last three decades (indicated with a * and where applicable cited below). The population estimates vary significantly, which may partially be explained by the lack of consensus regarding who is included in the Raute population. The population in our study is the one generally referred to as ‘Nomadic’. For some sources, it is unclear whether they inferred population sizes through direct counting or approximate estimates.

**Bibliography**

Paudel, M. K. (2016). *Resistance and change. A case study of economic changes and its effect on language, food habits and dress of the nomadic hunting-gathering Raute of Nepal* (Master's thesis, UiT Norges arktiske universitet).

Yasin, B. A. N. U. (2017). *The Raute community and the challenges to maintaining their indigenous ecological knowledge and practice* (Doctoral dissertation, M. Sc. Thesis. Graduate School of Life and Environmental Sciences, University of Tsukuba, Japan).
